# First record of *Isospora amphiboluri* in the thorny devil, *Moloch horridus*

**DOI:** 10.1016/j.ijppaw.2024.100983

**Published:** 2024-09-05

**Authors:** Katherine Adriaanse, Tamara Morgan, Robin B. Gasser, Anson V. Koehler

**Affiliations:** a Alice Springs Desert Park, Larapinta Drive, Flynn, 0871, Northern Territory, Australia; b Department of Veterinary Biosciences, Faculty of Science, The University of Melbourne, Parkville, Victoria, 3010, Australia

**Keywords:** *Isospora amphiboluri*, Coccidia, Reptiles, Thorny devil, *Moloch horridus*

## Abstract

Poor long-term survival (Mean = 2.16 y; 95% CI 1.68–2.65) was identified in a captive population of thorny devils (*Moloch horridus*) held at the Alice Springs Desert Park in the Northern Territory, Australia, over a period of 27 years. There was no significant difference in survival time (after acquisition) of wild-caught individuals compared captive born animals, or males compared to females. Limited information was available regarding the cause(s) of death for animals found dead or euthanased. Health of the live population at the time of the study (n = 14) was assessed by clinical history review, physical examination, and faecal examination. Large numbers of coccidian oocysts measuring 20–24 μm in diameter were identified upon faecal examination. Molecular investigation of genomic DNA from these samples identified *Isospora amphiboluri* based on the sequences of partial regions of the mitochondrial cytochrome *c* oxidase subunit 1 gene (*cox*1) and the nuclear small subunit of ribosomal RNA gene (*SSU*). *Isospora amphiboluri* was originally described from the bearded dragon (*Pogona barbata*) and has since been recorded in the inland bearded dragon (*Pogona vitticeps*) and the central netted dragon (*Ctenophorus nuchalis*). The present case expands the host range for *I. amphiboluri.* Histological examination of tissues was not available, and therefore the potential role of *I. amphiboluri* in morbidity and mortality of *M. horridus* is not clear. Further research is required to understand if colonization with *I. amphiboluri* is pathogenic in this species.

## Highlights

• Coccidia oocysts were detected in faeces of captive *Moloch horridus* (thorny devils).
• Coccidian species confirmed to be *Isospora amphiboluri* using molecular techniques.
• This report expands the known host range of *Isospora amphiboluri.*
• Clinical significance of *I. amphiboluri* infection in *M. horridus* not yet understood.

## Graphical abstract

**Figure:**
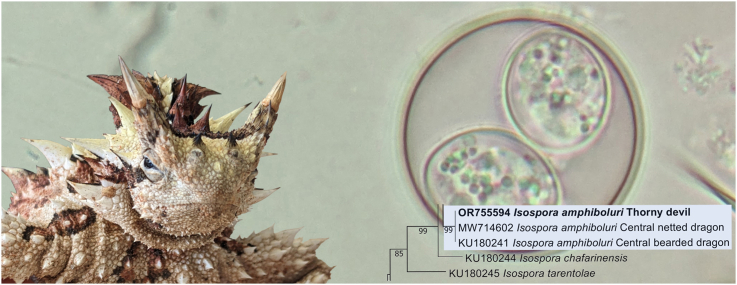

## 1 Introduction

*Isospora* is a genus of obligate intracellular apicomplexan parasites which infect the intestinal tract of a wide range of vertebrate hosts and are generally considered species-specific (Tenter et al., 2002; Morrison, 2009). Reptilian *Isospora* have diplosporocystic oocysts and are tetrasporozoic, yet are polyphyletic with amphibian, avian and mammalian *Isospora* species (Megía-Palma et al., 2016). *Isospora* species have been found in 11 major families of saurians (lizards), with 13 species described from the agamid lizards, commonly referred to as the ‘dragon lizards’ (Duszynski et al., 2008; Mihalca et al., 2009; Liu et al., 2021). In Australia, the agamid lizards belong to the subfamily Amphibolurinae (Pyron et al., 2013) from which two *Isospora* species have been described: *Isospora amphiboluri* (Cannon, 1967) and *Isospora cannoni* (Finkelman and Paperna, 1994). The oocysts and sporocysts of these two species are morphologically similar; however, the endogenous stage of *I. amphiboluri* is intracytoplasmic, whereas it is intranuclear for *I. cannoni* (Mihalca et al., 2009).

*Isospora amphiboluri* was first described by Cannon (1967) in an eastern bearded dragon (*Pogona barbata*; syn. *Amphibolurus barbatus*) from Queensland, Australia. It was re-described by McAllister et al. (1995) from the inland bearded dragon (*Pogona vitticeps*) and occurs most widely in this host species, which is important in the pet trade (Walden and Mitchell, 2021). *Isospora amphiboluri* has also been recorded in the central netted dragon (*Ctenophorus nuchalis*) (Liu et al., 2021).

*Isospora* of reptiles are known to cause diarrhoea, anorexia, weight loss and progressive apathy; disease is most common in juveniles, but individuals of all ages can be affected (Hallinger et al., 2019). The pre-patent period of *I. amphiboluri* is 15–22 days, with infection progressing from the duodenum to the colon (Walden and Mitchell, 2021). The anticoccidial toltrazuril is typically used for the treatment of infected reptiles (Hallinger et al., 2019).

The thorny devil, *Moloch horridus,* is a small myrmecophagous lizard, endemic to arid Australia. The species belongs to the family Agamidae and is the single member of the monotypic genus, *Moloch.* Due to its unique dietary and husbandry requirements, this species is rarely kept in captivity, and there is a paucity of health data pertaining to *M. horridus* in the literature.

Here, we report a case of infection by *I*. *amphiboluri* in a captive population of thorny devils at the Alice Springs Desert Park, Northern Territory, Australia. This case expands the host range of *I*. *amphiboluri* and challenges the preconception that *Isospora* species are host-specific (McAllister et al., 1995; Greiner, 2003; Megía-Palma et al., 2016).

## 2 Materials and methods

### 2.1 Study population

A captive population of thorny devils has been maintained at the Alice Springs Desert Park (ASDP), Northern Territory, since 1996. This population consists of wild caught individuals, animals rescued by wildlife rehabilitators and members of the public, and those bred in captivity at the institution. There has been continuous supplementation of the captive population with animals from the wild, with minimal quarantine screening and biosecurity measures in place prior to the introduction of new animals. At the time of this study, the live population consisted of 14 individuals. This population is housed in two groups: (1) a small indoor display enclosure, which houses a breeding pair and any current offspring, and (2) a large outdoor enclosure, which is walled and has a mesh roof. The outdoor enclosure is not accessible to wild thorny devils but may be accessible to other wildlife, including rodents, small reptiles and invertebrates. There is frequent movement of animals between the two enclosures. Prior to the date of this study (∼1 year), central military dragons (*Ctenophorus isolepis*) were also housed in the indoor enclosure with the thorny devils.

### 2.2 Historical case review and clinical diagnostics

In response to concerns about longevity of thorny devil individuals in the captive population, a review of current and historical records was conducted. Data analyses were performed using Stata (StataCorp, College Station, Texas, USA). Routine health checks of all live animals, including clinical history review, conscious physical examination and faecal examination were also performed. Faeces were collected from the enclosure substrate and pooled for both the indoor and outdoor enclosures. An individual sample was collected from an adult female thorny devil with loose faeces by direct observation. Routine faecal flotations were performed using a commercially available sodium nitrate flotation solution of a specific gravity of 1.2 (Dechra Veterinary Products, Somersby, New South Wales, Australia); faeces were suspended in sodium nitrate solution using a Fecalyzer® kit (Vetoquinol, Hamilton, Queensland, Australia), a cover slip was placed over the top of the Fecalyzer, and samples were left at room temperature for 10 min. Coverslips were then removed, placed on a glass slide and examined for endoparasites at 40-, 400- and 1000-times magnification by light microscopy.

### 2.3 Sample collection for molecular analysis

To specifically identify and genetically characterise the coccidian species infecting the thorny devils, samples were collected for molecular analysis. Faeces were collected from the substrate of the indoor enclosure and pooled. An additional sample was collected from the isolated female individual after treatment with toltrazuril (Baycox™, Bayer Australia and New Zealand, Pymble, New South Wales, Australia). For comparison, a pooled sample of faeces was also collected from a separate institution housing a population of thorny devils. There was no movement of animals between the two institutions. No demographic or health data were available about this population. Faeces were refrigerated for up to 48 h prior to examination, and a repeat faecal examination was performed (sub-section 2.2). A portion of each sample was preserved in 70% ethanol for subsequent DNA analysis (potassium dichromate was not available at the time of collection).

### 2.4 DNA extraction, PCR, sequencing

Ethanol-fixed faecal samples were washed three times with H_2_0 and allowed to rehydrate. Then genomic DNA was extracted from 250 μl of this faecal suspension using the DNeasy PowerSoil Pro Kit (cat. no. 47016, Qiagen, Venlo, The Netherlands) following the manufacturer's instructions.

DNA from each sample was subjected to PCR employing two genetic markers – mitochondrial cytochrome *c* oxidase subunit 1 gene (*cox*1) and nuclear small subunit of ribosomal RNA gene (*SSU*). A region (∼810 bp) of *cox*1 was amplified employing primers KM204 (forward: 5′- GTT TGG TTC AGG TGT TGG TTG -′3) and KM205 (reverse: 5′- ATC CAA TAA CCG CAC CAA GAG -′3) (Schwarz et al., 2009) using the following cycling protocol: 94 °C for 5 min (initial denaturation), followed by 35 cycles of 94 °C for 30 s (denaturation), 55 °C for 45 s (annealing) and 72 °C for 45 s (extension), with a final extension of 72 °C for 5 min. A partial region (∼1500 bp) of *SSU* was amplified employing primers EIF1 (forward: 5′- GCT TGT CTC AAA GAT TAA GCC -′3) and EIF3 (reverse: 5′- ATG CAT ACT CAA AAG ATT ACC -3′) (Yang et al., 2012) using the following cycling protocol: 94 °C for 5 min (initial denaturation), followed by 35 cycles of 94 °C for 30 s (denaturation), 60 °C for 45 s (annealing) and 72 °C for 90 s (extension), with a final extension of 72 °C for 7 min.

All PCRs were conducted in a volume of 50 μl including 2 μl of genomic DNA, the GoTaq Flexi buffer (Promega Australia, Alexandria, New South Wales, Australia), 3.0 mM of MgCl_2_, 200 μM of each deoxynucleotide triphosphate (dNTP), 25 pmol of each primer and 1 U of GoTaq DNA polymerase (Promega). Known test-positive (apicomplexan DNA), test-negative and no-template controls were included in each PCR run. The intensity and size of all amplicons were assessed by standard agarose electrophoresis (Koehler et al., 2016). Subsequently, PCR products were treated with the enzymes *Exo* I (Thermo Fisher Scientific, Waltham, Massachusetts, USA) and a thermosensitive alkaline phosphatase (FastAP, Thermo Fisher Scientific, USA) to remove primers and then sequenced bi-directionally using a standard protocol (Koehler et al., 2016).

### 2.5 Phylogenetic analysis of sequence data

The *SSU* (1427 bp; GenBank accession no. OR755594) and *cox*1 (787 bp; GenBank accession no. OR760030) sequences were compared with respective sequences in the GenBank database using the Basic Local Alignment Search Tool (BLAST; www.ncbi.nlm.nih.gov, Accessed October 2023) and then aligned with reference sequences representing distinct apicomplexan species and selected outgroup taxa from this database. Sequences were compared in a pairwise manner, and sequence identities recorded using Geneious Prime v. 2023.2.1 software (www.geneious.com, Accessed October 2023). Subsequently, sequences were aligned using the program Muscle (Edgar, 2004) and alignments adjusted manually using the program Mesquite v.3.81 (Maddison and Maddison, 2023). Subsequently, phylogenetic analyses were conducted using the neighbour-joining (NJ) distance method (Saitou and Nei, 1987) in the program MEGA v.11.0.1 (Tamura et al., 2021). Evolutionary distances were computed using the ‘number of differences’ method (Nei and Kumar, 2000), including ‘transitions and transversions’ for the nucleotide data. Rates of evolution among sites were considered uniform and gaps were treated using pairwise deletion. A total of 2000 bootstrap replicates were performed and are reported as bootstrap support percentages (bs). *Goussia* species were used as outgroups in the analyses.

## 3 Results

### 3.1 Historical case review and clinical findings

Records were available for 95 individual *M. horridus* housed at ASDP between 1996 and 2023. Of these animals, 52 were hatched at ASDP and 43 animals were wild caught. Most animals acquired from the wild were donated by members of the public or wildlife rescue groups. A total of 67 animals were reported as found dead or euthanased; 14 were missing, presumed dead; and 14 individuals comprised the live population (Table 1). Mean survival time after hatching or acquisition from the wild was 2.16 years (95% confidence interval 1.68–2.65 years; n = 95) and maximum survival time was 9.7 years. There was no significant difference in survival time (after acquisition) of wild-caught individuals compared to those hatched at ASDP (p = 0.28, two-sample *t*-test). There was no significant difference in survival time of males compared to females (p = 0.49, two-sample *t*-test). Weight loss prior to death was reported in 26.9% (18/67) of deceased animals, no other clinical signs were reported. Gross necropsy examinations were performed for 13.4% (9/67) of animals found dead or euthanased; no definitive cause of death was determined from gross necropsy for any case. Histopathology was available for one female found dead in 2021. The cause of death was thought to be due to a bacterial enteritis, although delay from death to post-mortem evaluation complicated histological interpretation. There was no histological evidence of metazoan or protozoan organisms in the gastrointestinal tract of this animal.

**Table 1:** Summary of outcomes for all thorny devil (*Moloch horridus*) individuals held in captivity at Alice Springs Desert Park between 1996 and 2023.

Outcome	Number of animals
Found dead	58
Euthanasia	9
Missing, presumed dead	14
Alive	14
**Total**	95

A physical examination was performed on all 14 live individuals. No clinical abnormalities were identified. One adult female was reported to have intermittent loose faeces, lethargy, and weight loss. No other individuals had any significant medical history.

### 3.2 Detection of coccidia in thorny devils

**Fig. 1:**
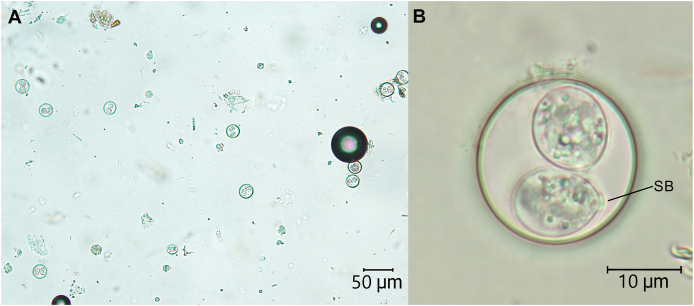
Photomicrographs of *Isospora amphiboluri* oocysts detected on examination of a pooled faecal sample from the captive thorny devil *(Moloch horridus)* population at Alice Springs Desert Park. (A) 400× magnification, scale bar: 50 μm; (B) High-magnification photograph of oocysts in (A); scale bar: 10 μm. SB = Stieda body.

Large numbers of coccidian oocysts were identified in all faecal samples collected from thorny devils housed at ASDP – the pooled sample from the outdoor enclosure, pooled sample from the indoor enclosure, and the sample from the female individual with intermittent loose faeces (Fig. 1; Table 2). Oocysts were spherical to subspherical and smooth-walled, with a diameter of 20–24 μm. Sporocysts were ellipsoidal, with a flattened Stieda body at one end. As the significance of the coccidial infection and the safety of chemotherapy with toltrazuril was unknown for this animal species, only the adult female with clinical signs was treated. The female was isolated from the group during treatment and subsequent faecal testing. Toltrazuril was administered orally at 20mg/kg once daily for 2 days. Approximately three weeks after treatment, a faecal sample was obtained from this individual for another faecal flotation. No oocysts were detected by light microscopy in this sample. The clinical signs of intermittent loose faeces resolved, but the lethargy and weight loss did not.

**Table 2:** Demographic data, conventional faecal examination and *cox1*-PCR test results for pooled and individual captive thorny devil (*Moloch horridus*) faecal samples collected in June 2023 in Alice Springs, Northern Territory.

Sample no.	Type	Enclosure	Demographics	Faecal examination (flotation/microscopy)	*cox*1-PCR test result
1	Individual	Alice Springs Desert Park – Indoor Isolation	1 adult female	Negative for coccidia oocysts^a^	Negative
2	Pooled	Alice Springs Desert Park – Indoor Group	2 adults, 3 juveniles	Positive for coccidia oocysts	Positive
3	Pooled	Alternative institution	Not known	Negative for coccidia oocysts	Negative

a After treatment with toltrazuril at 20 mg/kg orally, once daily for two days.

No oocysts were detected by faecal flotation/microscopy in the pooled faecal sample from the alternative institution (sample 3; Table 2). This sample, the sample from the treated female (sample 1; negative for oocysts), and a repeat pooled sample from the indoor enclosure (sample 2; confirmed to still be positive for oocysts on faecal flotation) were submitted for molecular analysis. PCR amplified *cox*1 and *SSU* exclusively from sample 2 (Table 2).

### 3.3 Genotyping and phylogenetic trees

**Fig. 2:**
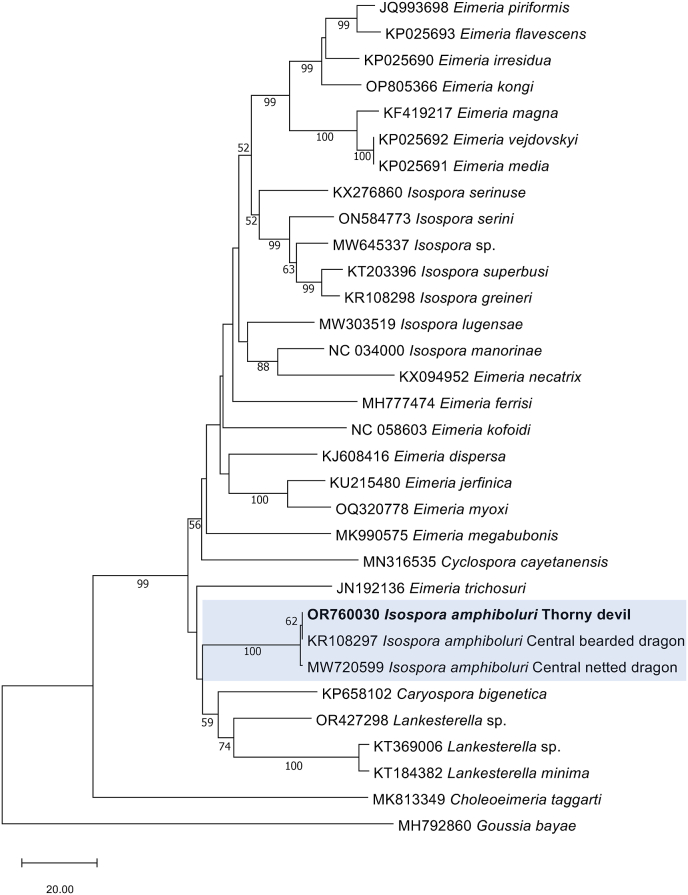
Phylogenetic relationship of *Isospora amphiboluri* (in bold) from pooled thorny devil faeces with representative coccidian sequences, established based on an analysis of sequence data from a 760 bp portion of the mitochondrial cytochrome *c* oxidase subunit 1 gene (*cox*1) employing the neighbour-joining distance method. Branch supports are represented by neighbour-joining bootstrap percentages. *Goussia bayae* was used as an outgroup.

**Fig. 3:**
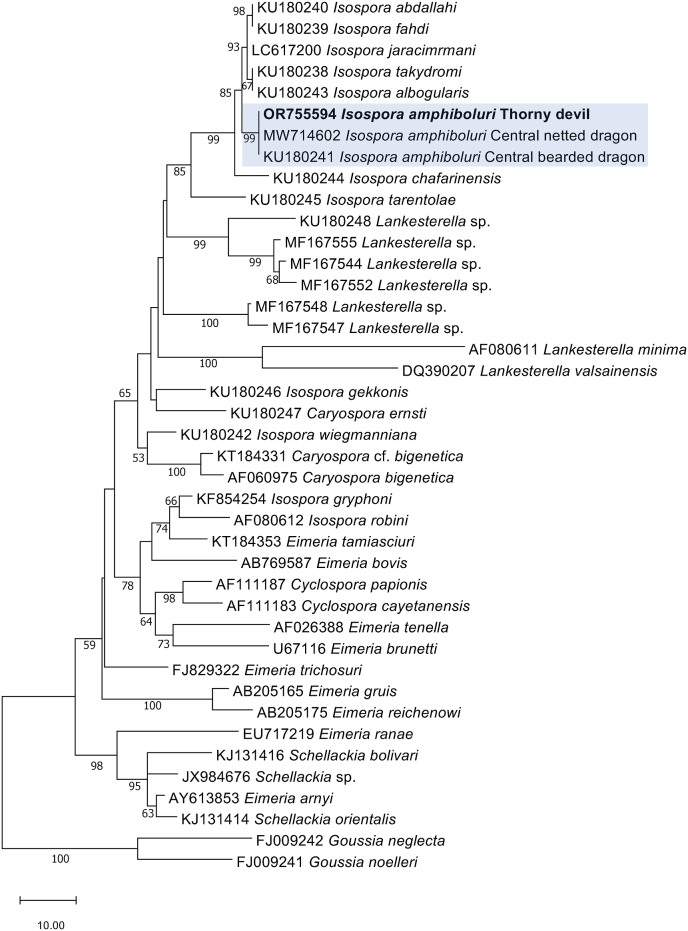
Phylogenetic relationship of *Isospora amphiboluri* (in bold) from pooled thorny devil faeces with representative coccidian sequences, established based on an analysis of sequence data from a 1427 bp portion of the nuclear ribosomal small subunit of RNA gene (*SSU*) employing the neighbour-joining distance method. Branch supports are represented by neighbour-joining bootstrap percentages. *Goussia neglecta* and *G. noelleri* were used as outgroups.

The sequences obtained were compared with those in the NCBI database using BLASTn. The *cox*1 sequence (GenBank accession no. OR760030; 787 bp) matched exactly with the sequence representing *I. amphiboluri* (GenBank accession no. KU180297) obtained from an inland bearded dragon (*P. vitticeps*) in Canada (Hafeez and Barta, 2019). Similarly, the *SSU* sequence comprising 1427 bp (GenBank accession no. OR755594) was identical to that representing *I. amphiboluri* (GenBank accession no. KU180241) from an inland bearded dragon in Spain (Megía-Palma et al., 2016). The phylogenetic tree constructed with the *cox*1 sequence of *I. amphiboluri* from the thorny devil along with closely related reference sequences from GenBank, using *Goussia bayae* as an outgroup, had a well-supported clade of *I. amphiboluri*; however, no sequences from alternative *Isospora* species infecting reptiles were available for *cox*1 (Fig. 2). When the *SSU* sequence of *I. amphiboluri* from the present thorny devil was placed in a phylogenetic context with closely related reference sequences from GenBank, using *Goussia* sp*.* as outgroups, there was a well-supported clade of *I. amphiboluri* grouping with other *Isospora* species found in reptiles (Fig. 3).

## 4 Discussion

In this study, we identified and genetically characterised *I*. *amphiboluri* from a group of captive thorny devils with poor long-term survival. The unsporulated oocysts were initially thought to be *Eimeria molochis*, described previously for the thorny devil (Bovee and Telford S.R., 1965); potassium dichromate was unavailable at the time of this study and therefore oocysts were not successfully sporulated. However, identical sequence matches for *cox*1 and *SSU* confirmed the specific identification as *I. amphiboluri*.

*Isospora amphiboluri* is most often reported from captive inland bearded dragons (*P*. *vitticeps*), and in some studies the prevalence is quite high. Three independent studies of captive agamid lizards in Germany highlighted a notable prevalence of *Isospora* sp. infections ranging from 17% (127/747) (Hallinger et al., 2019) to 28.7% (337/839) (Stöhr et al., 2021) and up to 43.5%, (168/386) (Pasmans et al., 2008). The two former studies suggested that the species was *I. amphiboluri*, whereas the latter reported *Isospora* sp.

McAllister et al. (1995) inferred that Australian coccidia are either species or genus specific and that they do not cross generic and familial host boundaries. Furthermore, Megía-Palma et al. (2016) stated that *Isospora* species that infect lizards show a high degree of host specificity, as evidenced by the high diversity of species described in reptiles. Unfortunately, molecular characterisation to confirm the identity of *Isospora* species reported in agamid lizards is rarely performed, with the first sequences of *I. amphiboluri* deposited in GenBank in 2016 (Megía-Palma et al., 2016). It should be noted that the agamid host species from which *I. amphiboluri* is described*,* i.e. the central bearded dragon (*Pogona vitticeps*) (McAllister et al., 1995), eastern bearded dragon *(Pogona barbata)* (Cannon, 1967)*,* central netted dragon (*Ctenophorus nuchalis*) (Liu et al., 2021), and now the thorny devil *(Moloch horridus)* (this study)*,* are all endemic to Australia and are sympatric within their distributions. It is therefore possible that all records of *Isospora* in agamid lizards might be the same species, but this requires molecular confirmation. Specificity at the host genus or family level, rather than species, has been observed with *Isospora* of passerine birds (Berto et al., 2011), and may also be the case for reptilian *Isospora*. Regardless, future work on *Isospora* in reptiles should include molecular confirmation of species.

In this report, histological samples showing colonization of the intestinal epithelium were not available and, therefore, we cannot definitively confirm a patent infection versus gut passage of oocysts. However, given that these animals were all housed in single-species groups for >12 months without access to other agamid species, true infection is likely.

The significance of infection with *I. amphiboluri* in thorny devils is unclear. The study population had a mean survival time of 2.16 years, which suggests markedly reduced longevity when compared to reported longevity of wild thorny devils of 6–20 years (Pianka and Pianka, 1970). Unfortunately, the lack of clinical surveillance and histopathological data for this population meant that the possible contribution of *I. amphiboluri* infection to reduced lifespan was not able to be determined. One infected adult female showed intermittent loose faeces, lethargy and weight loss, but only the abnormal faeces resolved after treatment with toltrazuril. A possible explanation for this is the presence of co-morbidities contributing to the weight loss and lethargy observed in this individual. Infection was identified in clinically normal individuals; however, subtle changes are difficult to detect in this species and, therefore, early or mild signs of disease may have been missed. More investigation is needed to understand the clinical significance of *I. amphiboluri* infection in thorny devils.

## CRediT authorship contribution statement

**Katherine Adriaanse:** Writing – review & editing, Writing – original draft, Project administration, Investigation, Formal analysis, Data curation, Conceptualization. **Tamara Morgan:** Writing – review & editing, Investigation, Conceptualization. **Robin B. Gasser:** Writing – review & editing, Supervision, Project administration. **Anson V. Koehler:** Writing – review & editing, Supervision, Resources, Methodology, Formal analysis.

## Declaration of competing interest

The authors declare no conflict of interest. We wish to confirm that there are no known conflicts of interest associated with this publication and there has been no significant financial support for this work that could have influenced its outcome. We confirm that the manuscript has been read and approved by all named authors and that there are no other persons who satisfied the criteria for authorship but are not listed. We further confirm that the order of authors listed in the manuscript has been approved by all of us. We confirm that we have given due consideration to the protection of intellectual property associated with this work and that there are no impediments to publication, including the timing of publication, with respect to intellectual property. In so doing we confirm that we have followed the regulations of our institutions concerning intellectual property. We understand that the Corresponding Author is the sole contact for the Editorial process (including Editorial Manager and direct communications with the office). She is responsible for communicating with the other authors about progress, submissions of revisions and final approval of proofs. We confirm that we have provided a current, correct email address which is accessible by the Corresponding Author.
